# Linking the choice of the class format and preclass learning experiences sheds light on a step further in blended medical education

**DOI:** 10.1080/10872981.2023.2186207

**Published:** 2023-03-03

**Authors:** Cheng-Maw Ho, Chi-Chuan Yeh, Jann-Yuan Wang, Rey-Heng Hu, Po-Huang Lee

**Affiliations:** a Department of Surgery, National Taiwan University Hospital, Taipei, Taiwan; b School of Medicine, College of Medicine, National Taiwan University, Taipei, Taiwan; c Department of Internal Medicine, National Taiwan University Hospital, Taipei, Taiwan; d Center of Faculty Development and Curriculum Integration, College of Medicine, National Taiwan University Hospital, Taipei, Taiwan; e Department of Surgery, E-Da Hospital, I-Shou University, Kaohsiung, Taiwan

**Keywords:** Online, HyFlex, synchronous, blended learning, class format preference

## Abstract

**Background:**

The core principle of HyFlex (‘hybrid’ and ‘flexible’) learning is to maintain learning equity under most circumstances. Within a blended framework in precision medical education, how different preferences of synchronous learning environment influence learning process and outcome is limited. We investigated students’ preclass online video learning experiences and their choices toward synchronous class formats.

**Methods:**

This was a mixed-methods study. During the 2021 academic year, all 5th-year medical students who had viewed online video clips presenting core concepts were asked to complete a survey on their preference for future synchronous class format (face-to-face, online, or HyFlex) and asked to provide reflective comments on their self-learning. Anonymous survey data, online records, and summative assessment scores (short-term learning outcomes) were collected. Kruskal – Wallis or Chi-square tests were used to compare differences between groups, and multiple linear regression was managed to select the factors associated with various choices. The students’ comments were coded in a descriptive thematic analysis.

**Results:**

Among 152 medical students, 150 responded to the questionnaires, and 109 provided comments. Medical students spent a median of 32 min online, significantly shorter in the face-to-face group than in the online and HyFlex groups. The online group had a lower preclass video completion rate for certain concepts. The choice was not associated with short-term learning outcomes. Student feedback revealed a higher frequency of multiple themes for each student in the face-to-face and HyFlex groups, and these themes fell into the categories of learning efficiency, focus concentration, and course attractiveness.

**Conclusions:**

Linking the choice of the class format and learning experiences of preclass online videos sheds light on a step further within a blended framework of precision medical education. Supplement of online interactive elements may help secure learning engagement among students choosing ‘online only’ class format of HyFlex learning.

Online learning in medical education has become increasingly popular during the COVID-19 pandemic [1–3]. However, medical science is a complex discipline that requires coaching, tutoring, and interactive concept clarification for the student to attain a complete grasp of the subject. Online learning may threaten inequities of learning opportunity and the quality of learning in medical education because the access and the online content may be different from real-world class, which may compromise the quality of the future medical care. Therefore, pedagogical methods in the context of online learning must be formulated.

Learning climate is defined by Seif et al. as ‘the social, emotional, and physical conditions under which one acquires knowledge.’ [4] Conventional classroom or environment (within the hospital) climate is an essential factor for learning, student satisfaction, and reduced distraction [5–9]. Climate is a key topic in online education because the setting can significantly vary depending on the local environment surrounding the learners [10–14]. Although synchronous online learning can be recorded for learners to play back anytime, it has the same concern regarding learning environment as asynchronous online learning. We understand that a learner would choose a class format, either online or face-to-face, that hosts one’s favored learning environment with a positive climate supporting one’s learning [15].

Other factors that students prefer online learning to face-to-face class included flexibility of time [16] and less confrontational nature [17]. In higher education, students who prefer face-to-face class may worry that online learning would be insufficient of teacher explanation and interaction and would be with weaker student-student interaction [18]. As a result, students did not want to risk taking difficult courses online and preferred what they considered to be the richer experience of the face-to-face classroom [18]. Consistently, when conceptual knowledge in the subject matter or skills in the application of one’s knowledge are to be acquired, students may prefer face-to-face learning [19]. However, when skills in self-regulated learning are to be acquired, students advocate online learning [19]. Online and face-to-face activities can lead to similar levels of academic performance, and students would rather do written activities online but engage in discussion in person [20]. We can summarize that class format preference is related to class content and learning environment. Thus, class format preference plays a role in learning process and the quality of learning process may be threatened in students who choose online format in medical education.

The HyFlex (‘**hy**brid’ and ‘**flex**ible’) model, proposed by Beatty, is an instructional approach that adapts face-to-face and online learning (synchronous online [live broadcast] and asynchronous online) [21]. The HyFlex course design is built upon on four pillars: learner choice (provide meaningful alternative participation modes and enable students to choose between participation modes daily, weekly, or topically); equivalency (provide learning activities in all participation modes which lead to equivalent learning outcomes); reusability (utilize artifacts from learning activities in each participation mode as ‘learning objects’ for all students); accessibility (equip students with technology skills and equitable access to all participation modes) [21]. The core principle of HyFlex learning is to maintain learning equity under most circumstances, either in-person, synchronously online, or asynchronously online [21]. HyFlex learning in medical education is a flexible alternative during the pandemic. A well-designed HyFlex class, with effective alternative participation modes that lead to the same learning outcomes, can provide meaningful learning opportunities for students [22]. However, studies on higher education have reported numerous problems in the process of adapting to the hybrid teaching model, including decreased motivation, loneliness, technical connection problems, and less interaction with the teaching staff and other students [23,24]. With regard to medical education, a study conducted in Kings College reported that HyFlex seminars entail a higher cognitive load than face-to-face classes and may not suit everyone and every teaching session [25]. These experiences indicate the difficulty of creating a successful online learning adaptation. Research investigating medical students’ preferences to the HyFlex learning model is limited, and we should therefore consider their decisions and reduce the online learning barriers to ensure that HyFlex classes are implemented well.

## Theoretical background

Knowles defined self-directed learning as ‘a process by which individuals take the initiative, with or without the assistance of others, in diagnosing their learning needs, formulating learning goals, identifying human and material resources for learning, choosing and implementing appropriate learning strategies, and evaluating learning outcomes’ [26]. To facilitate self-directed learning efficiency and to incorporate online learning into consideration, attempts had been made to develop instruction models that support learning process other than the learning content [27]. We previously reported practicing precision medical education based on cognitive load and multimedia learning theories [28–32]. We theorized that practicing precision into blended medical education is feasible with asynchronous online video learning because it can strike a balance between ever-increasing cognitive load of the class content and ensuring that students can engage in the embodied practice necessary in precision medical education [28]. Further, we would like to access students’ choices of synchronous class format as the preferred learning environment, in hope to get clues of adjusting teaching strategies and facilitate learning process in their preferred class formats.

Regular university or hospital class operations can be threatened by many factors, including climate change, natural disasters, and public health crises such as the COVID-19 pandemic [33]. Instructors using HyFlex can ensure instructional continuity during such disturbances [33]. The present state of medical teaching highlights the need to initiate a practical HyFlex class format under a blended learning structure because medical teaching relies heavily on online learning and online video materials [34].

Supplementary Figure S1 summarizes the interrelation of concepts (learning environment and online video learning with reduced cognitive load [28] and study context. By investigating the link between the choice of the synchronous class format and the learning experiences of previous visualization of preclass videos, we may get clues of adjusting teaching strategies to enhance individualized learning efficiency in medical education.

Within a blended framework in precision medical education, how different preferences of synchronous learning environment (including consideration of HyFlex learning) influence learning process and outcome is not well addressed. We hypothesized that medical students who chose the synchronous class format (face-to-face, online, or HyFlex [both are good, with flexibility]) might have different short-term learning outcomes and associated features of preclass online video learning. Therefore, this study examined students’ choices toward synchronous class formats after preclass online video learning and investigated the associated factors.

## Methods

### Research type and participants

**Figure 1.**
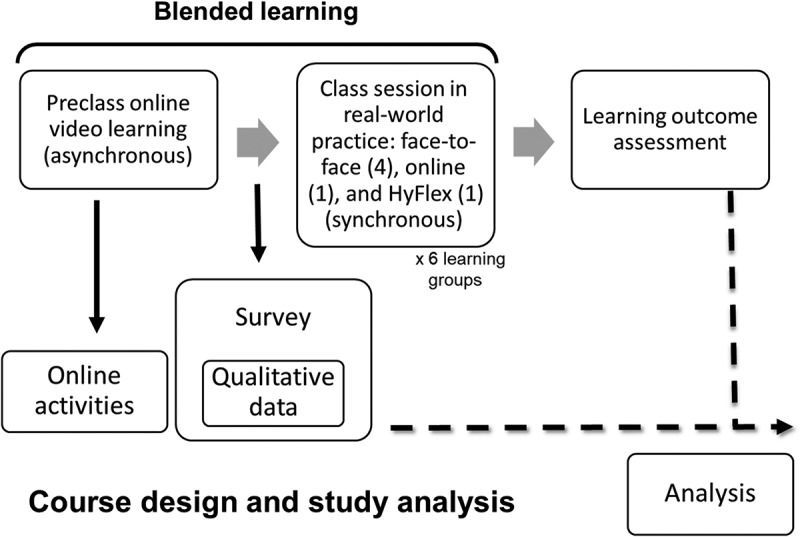
Course design and study analysis. A total of six rounds (learning group 1–6) was conducted in one academic year. Each course section comprised a 1-hour class (synchronous learning), with 25–27 medical students enrolled in each round. Preclass online video learning included six video clips, each lasting less than 2 min, for six individual concepts were made to minimize the extraneous cognitive load. A survey was then asked on their preference of learning environment for future synchronous class format (face-to-face, online, or HyFlex [both are good]) and to provide reflective comments on their self-learning. Note that the real-world class format was implemented according to the regulations of that time for social distancing in the classroom and not their choices. The eventual class formats in 6 learning groups were 4 face-to-face (relaxed regulation), 1 online (stricter regulation), and 1 HyFlex (strict regulation). Online activities included time spent for watching the videos, the number of webpage visits, total time spent online, and completion rates of watching each video. Preclass online video learning experiences, including survey data and online activities records, and short-term learning outcome were analyzed. A total of 150 participants were enrolled in the study.

This cross-sectional mixed-methods study (Figure 1) [34,35] administered a survey with scale-based (quantitative) questions and open-ended (qualitative) sections where respondents could provide comments [34]. The topic of acute liver failure constituted a section of a compulsory core course for surgery students in their fifth year of university [34]. Almost all students were 23 − 24 years of age. Each course section comprised a 1-hour class, with 25–27 medical students enrolled in each round. A round in surgery takes 6 weeks. A total of six rounds (learning group 1–6) is conducted in one year. Each student was randomly allocated by medical school into one of the six learning groups. Between September 2021 and May 2022, 159 fifth-year medical students took the course and were asked to participate in this survey. The Institutional Review Board of National Taiwan University Hospital approved this study as an exempt protocol (201809078W and 202006048W). Participation in the survey was considered to imply consent.

### Course design and practice

The curriculum development committee assigned Cheng-Maw Ho (HCM) to develop the course section of acute liver failure. Because the allotted official time for this course section in each round is only 1 hour for this topic, the teacher had to adopt strategies to cope with the time shortage and to fill up individualized learning gaps as much as possible. HCM summarized six threshold concepts of acute liver failure (details in reference 28) according to the educational goals and integrated them into the course design and practice [28,34,36]. A blended course section on acute liver failure has been implemented since 2018 [28,34,36].

The course proceeded in two stages, the first comprising preclass online video learning and the second comprising face-to-face classroom instruction. Since 2021, social distancing measures in the classroom varied in severity depending on the state of the COVID-19 pandemic. For example, students were potentially barred from face-to-face classes if they were recently in close contact with someone with COVID-19. HyFlex instruction was then adopted if face-to-face learning was not allowed. The instructional methods for the online video portion of the course were based on the principles of coherence (excluding extraneous learning content) and segmenting (content is presented at the user’s own pace rather than as an unpausable video) in Mayer’s theory of multimedia learning [31,32].

Previously, six video clips, each lasting less than 2 min, for six individual concepts were made to minimize the extraneous cognitive load [28,34]. A eye-catching title with a summary of each concept was added. The clinical teacher (HCM) uploaded the updated review literature selectively to the webpage for reference reading, which supplemented further self-learning if students want to know more about the topic. Cases were selected on the intranet for clinical case analysis. As part of the short-term outcome assessment, each student had to select one clinical case and do critical analysis based on what they had learned.

### Online survey

At the start of the surgery course, students were instructed to watch the online videos in advance. Then, they were free to respond to an online questionnaire (details in reference 22, also in supplementary Table S1) on the intranet [37]. A validated questionnaire in English consisting of 12 items (without sub-dimensions) [28], with a mixture of question styles including 5-point Likert-type questions, was used to collect the data. The reliability of the questionnaire is assured by parallel forms method previously [36]. Additional question, ‘if conditions permit, the preferred class format for the upcoming class is: face-to-face, online, or HyFlex “both are good”’, was added to the online questionnaire. The categories that were evaluated pertained to the following: degrees of concept agreement after pre-class online video learning, compared to the understanding before learning ranging from 1 (totally disagree) to 5 (totally agree); concepts that needs more instructions in the coming class; concepts that do not need further instructions in the upcoming class; concepts that the student had learnt most; satisfaction toward the use of online videos prior to class; preference in class format (face-to-face, online, or HyFlex) for the upcoming class; and student comments or questions. The teacher then developed the major content of the upcoming synchronous class for each round of students based on the survey responses, aiming to fill up learning gaps and towards precision medical education [28].

## Objective assessment of short-term outcome

The outcome measurements in this study consisted of two summative assessments concerning the acute liver failure section, as previously described [34].

### Online learning activities

Cumulative website page views and webpage visit/browsing durations for each medical student were documented anonymously. Video completeness was calculated automatically by the ratio of duration a video was played for to the whole video duration. Fast forwarded, rewound, or skipped segments were not included in the calculation.

### Data collection

For each round of students, an administrative teaching assistant (SKW) helped collect anonymous survey data, demographic data, scores, and information on online activities.

### Qualitative data analysis

Student comments were independently coded through a descriptive thematic analysis by a specialist surgeon (HCM) and an administrative researcher (WJY), using Braun and Clarke reference [28,34,38]. Code management policy was described previously [34]. In brief, a team comprising a surgeon specialist (HCM), an experienced medical education specialist (YCC), and an administrative researcher (WJY) would discuss and resolve all coding discrepancies and to combine codes in regular meetings [28]. According to the survey responses in each round of students, the teacher in charge (HCM) answered questions posed by students, and adjusted the class content weight [28].

At the end of the school year, a final set of identified themes were generated to represent the range of student feedback on the learning process [34]. Research questions were used to collect student thoughts on the self-reported learning process and experiences with course framework design, particularly their choices towards face-to-face and online learning. Each student comment may include several codes across various categories (general, learning, course design, feeling and expectation, and miscellaneous).

### Quantitative data analysis

Quantitative data are expressed in terms of the mean, median, or percentage. Non-parametric tests (the Kruskal – Wallis or Chi-square tests) were employed to compare group differences. A multiple general linear regression model was managed to select potential factors associated with class format preferences using a backward elimination method. A two-sided *P* < .05 was considered statistically significant. Statistical analyses were performed using SPSS version 21.0 (SPSS, Chicago, IL, USA).

## Results

### Demographics and class format preference

Of the 152 fifth-year medical students (152/159, 95.6%) who participated in online video learning and completed the survey about future class format preference, 150 were enrolled in the study, excluding 2 who preferred no further classes. The participants were predominantly male (72%), were satisfied with online learning (96.0%), and provided comments (72.7%; Table 1). In the summative assessment of short-term learning outcomes, the average scores were 90.6 ± 8.5 and 2.2 ± 1.0 for the clinical case-based analysis and for the essay question, respectively (Supplementary Table S2).

**Table 1.** Student demographics and characteristics of preclass online video learning process.

	All	Face-to face	Online	HyFlex	*P*
n	150	71	42	37	
Male gender	72.0	70	71	76	0.675
Learning group					0.013
*1*	18.0	14	33	8	
*2*	18.0	21	10	22	
*3*	16.7	18	12	19	
*4*	16.0	18	12	16.	
*5*	14.7	6	17	30	
*6*	16.7	23	17	5	
Satisfaction (S)					0.321
*Strongly S*	52.7	59	48	46	
*S*	41.3	37	41	51	
*Average S*	6.0	4	12	3	
*Dis-S*	0	0	0	0	
*Strongly dis-S*	0	0	0	0	
Preclass video completed (SD)					
*C1*	96.0 (17.6)	99.3 (1.3)	90.2 (28.8)	96.3 (16.3)	0.174
*C2*	96.5 (16.6)	99.5 (1.1)	92.4 (26.0)	95.5 (18.2)	0.423
*C3*	96.3 (16.3)	99.4 (0.6)	91.4 (26.1)	96.0 (16.5)	0.071
*C4*	97.0 (16.2)	99.9 (0.4)	92.6 (26.1)	96.5 (16.6)	0.315
*C5*	96.1 (17.9)	99.4 (1.0)	90.1 (29.5)	96.7 (16.4)	0.444
*C6*	95.7 (18.0)	98.8 (3.7)	89.7 (29.5)	96.4 (16.3)	0.833
Providing comments	72.7	73	71	73	0.972
Total online time (min)*	32.0 (17.9–44.9)	24.7 (14.6–38.5)	38.1 (21.1–46.9)	37.1 (23.4–50.3)	0.007
Webpage visit counts*	146.0 (103.8–229.3)	147.0 (104.0–228.0)	137.5 (100.8–194.3)	161.0 (102.5–239.5)	0.734

Data were expressed in percentage unless otherwise mentioned. C, core concept; IQR, interquartile range; SD, standard deviation. *median (IQR).

In addition, 71 (71/150, 47.3%), 42 (42/150, 28.0%), and 37 (37/150, 24.7%) participants expressed a preference for synchronous face-to-face classes, online classes, and HyFlex classes, respectively. These preferences differed between learning groups (*P* = 0.013). Learning group 1 favored online classes (14/27), learning group 5 favored HyFlex equally (11/22), and learning group 6 favored face-to-face classes (16/25). There was no statistical difference in gender distribution among the 6 learning groups. Implemented according to the regulations for social distancing in the classroom, the eventual real-world class formats in each group were face-to-face (learning group 2–5, relaxed regulation), online (learning group 6, stricter regulation), and HyFlex (learning group 1, strict regulation). In learning group 1, 11 out of 27 students attended the class online with other classroom students using the Webex video meeting system.

Regarding the understanding of core concepts (C), C2, C3, C4, and C6 had neutral agreement with previous understanding; C4 and C6 were concepts that most students wanted to learn more about; C1 was the concept that most students suggested the teacher to talk less in class; and students exhibited improvements in their knowledge of C2, C3, C4, and C6 (more than 50%) (Supplementary Table S1). The distribution patterns for the themes of concept agreement, wishing to learn more about a concept, suggesting the teacher to talk less a concept in class, and exhibiting improvement in one’s understanding of a concept did not differ between the face-to-face, online, and HyFlex groups.

### Online video learning activities

Participants spent a median of 32 min online (interquartile range [IQR], 17.9–44.9 min) including time for watching the videos, and the median number of webpage visits was 146.0 (103.8–229.3). The online group spent the most time online (*P* = 0.007, Kruskal – Wallis test). All six videos had completion rates over 95%. However, the completion rate of the video for C3 (median 91.4%) was lowest in the online group (face-to-face group: 99.4%; HyFlex group: 96.0%) (*P* = 0.071, Kruskal – Wallis test). The three groups did not significantly differ in page visits, concept learning, satisfaction, and short-term learning outcomes.

### Factors associated with class format preference in multivariable analysis

A multiple general linear regression model was constructed with the increasing value of y toward online preference (y = 0, 1, 2, representing format choices of face-to-face, HyFlex, and online only, respectively). Longer total online time (coefficient [95% confidence interval], 0.000178 [0.000049–0.000308], *P* = 0.007) and less completion of the C1 preclass video viewing (−0.011 [−0.018- −0.003], *P* = 0.005) were associated with a preference for the online class format. Together, these objective data of preclass asynchronous online behavioral characteristics suggested students’ preference for upcoming synchronous class format. Attention might need be paid to students who prefer online learning in the design of HyFlex learning because we speculate that students choosing this preference tend to get distracted, paused, or did something else.

### Student feedback

**Figure 2.**
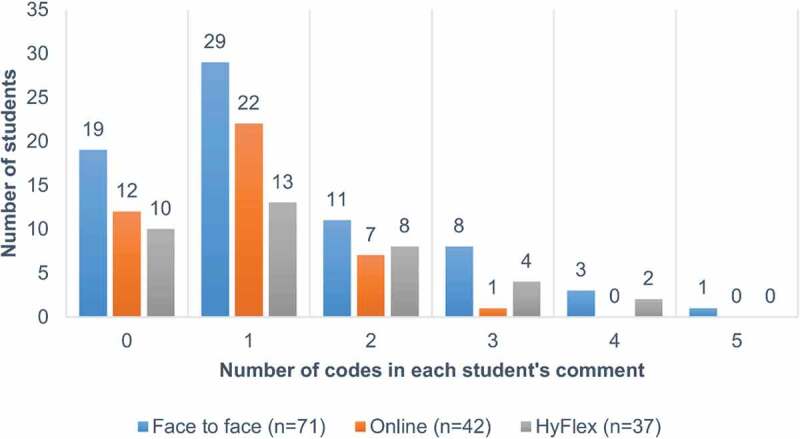
Distribution of code number in each student’s comment among the three groups of preference of learning environment for future synchronous class format (face-to-face, online, or HyFlex [both are good]). Multiple codes (code number≥3) per student were more prevalent in the face-to-face and HyFlex groups than in the online group (*P* = 0.068, Kruskal – Wallis test).

Table 2 presents student feedback after online video learning. The most common comments were coded in the category of ‘general: superficial or expressing gratitude,’ followed by ‘content, video-based course design and framework’ and ‘learning, conceptual correction, learning effect/efficiency.’ The themes in the categories ‘attractiveness and inspiration’ and ‘focused attention and concentration’ were not ever reported in the online group. Figure 2 presents the distribution of the codes (by code number) among the three groups. Among participants providing comments, multiple codes (code number≥3) per student were more prevalent in the face-to-face and HyFlex groups than in the online group (*P* = 0.068, Kruskal – Wallis test).

**Table 2.** Students’ feedback.

Coding category*	Code frequency (%)		
	Face-to-face	Online	HyFlex
Number of participants	71	42	37
General: superficial or expressing gratitude	38	38	35
*Example*	*Thanks for terrific videos (general, gratitude).*		
Specific learning content query	14	7	19
*Example*	*I would like the teacher illustrate how to approach a patient with suspected acute liver failure (query).*		
Learning, concept correction, effect, and efficiency	28	12	19
*Example*	*Clarify misunderstanding concept (correct concept); good learning efficiency and effect (learning, efficiency)*		
Content, video, course design & framework	28	24	27
*Example*	*Well-designed (design); quick to find where need to strengthen (framework); have motivation to finish the preclass content (content); don’t need to follow a long class with contents many of which already known (content, design).*		
Attractiveness & Inspiration	10	0	19
*Example*	*Concise videos and interesting titles attract me (attractiveness); not fall asleep while watching (attractiveness, focus); inspire me to think and look up more relevant information (inspiration).*		
Focused attention & concentration	4	0	5
*Example*	*Short videos make me focused easily (focus). Finish watching without knowing time had passed (focus); short videos can keep us in a high degree of concentration (concentration).*		
Class expectation: online or face to face	6	12	5
*Example*	*This preview can get us more into later face-to face class (face-to face, class expectation); expect to learn more in class (class expectation); really like online class for the easy access and can go back any time (online class).*		
Miscellaneous	1	0	0
*Example*	*Good narration (others).*		

*Examples of typical remarks and codes listed below.

## Discussion

Our study has four significant findings. First, the distribution pattern of students’ choice of class format was different between rounds. Second, participants who preferred the online class format spent more time online than the face-to-face and HyFlex groups, but some of the online group’s video completion rates were lower for specific topics, as indicated also in a multiple linear regression analysis. Third, participants who preferred online classes made open-ended comments that corresponded to fewer codes than the other two groups, where the number of codes for both groups were similar. Fourth, and finally, all three groups had similar short-term learning outcomes.

### Choosing “online”, attention, and short-term learning outcome

Our results showed that students who chose ‘synchronous online’ classes spent more time learning online but were less likely to complete the asynchronous preclass learning activity. We speculate that this is because students got distracted, paused, or did something else. Consistently, the themes in the categories ‘attractiveness and inspiration’ and ‘focused attention and concentration’ were not ever reported in the online group. Their preferences of class formats did not equal to the real-world class formats they received. Besides, during the synchronous class, the teacher would fill up the information gap discovered from the preclass surveys. All above may explain that the short-term learning outcome didn’t support the group difference, However, we cannot exclude the possibility that these students didn’t complete the topic because they already obtained key information. Although our observations on asynchronous learning may not directly generalize to future implementations of synchronous online learning, the behaviors reported in this study warrant further analysis. Surveys from UK medical students indicate that the most significant benefit of online teaching platforms is their flexibility and their most common barriers include distraction from family and a poor internet connection [39]. A conventional classroom format has the most rigid structure and helps students remain focused. Online student routines are likely more sedentary, and students may experience increased fatigue from prolonged screen use [40]. Effective breaks should include a change in posture to mitigate fatigue and promote concentration [40]. Mittal et al. detailed how remote learning can be made more effective through technology and open-access resources that encourage independent learning [41].

### Preferences enlighten teaching adjustment during the synchronous class within the framework of blended learning

We had ever practiced blended learning and HyFlex model with timely response to students’ still-existed knowledge gap in the following synchronous class smoothly. Learning experiences from asynchronous online video learning, by using a threshold strategy to reduce cognitive load on a precision medical education basis, achieved high student satisfaction and good short-term learning outcomes. In view of precision medical education, in-depth understanding of the link of students’ preferred class format with preclass videos learning may further help refine teaching strategies to supplement the need learners might not be aware of and to enhance student learning efficiencies. This sheds light on a step further in precision medical education. For example, supplement of interactive elements (discussion, graphs, animations, games, or timelines) in synchronous or asynchronous online learning process might help secure learning engagement among students choosing ‘online only’ class format.

The main difference between asynchronous and synchronous learning is that synchronous learning features live instruction occurring at a predetermined time [42]. Asynchronous online lectures with high cognitive intensity might not be an effective teaching format, especially during abrupt educational changes [43]. The synchronous method of online teaching was preferred to the asynchronous method by a majority of students in a medical laboratory technology course [44]. We argue that both synchronous and asynchronous learning have advantages and limitations. However, when combined, they can allow medical students to arrange their schedule more flexibly, enabling them to learn practical skills in face-to-face education in accordance with strict college/hospital learning protocols [45]. Instructors are responsible for designing a workable model that is adapted to medical students in an everchanging environment. When blended learning and HyFlex format were combined, together with on-time feedback of teaching adjustment strategies, sufficient learning equity is assured.

## Limitation

Asynchronous learning experience where students watch a video before a class may not share similar learning climates as synchronous learning. Although we provided the format options of HyFlex preference, we did not officially record the live broadcast and let students to learn by playing back. Different contextual elements related to COVID-19, such as predominate mutant strains and changing administrative regulation over time, might have a role in students’ preference of class format. But learning group, as a variable partly reflecting these elements, could not explain the preference after multivariable adjustment. Another limitation is that although short-term learning outcomes are similar among these highly selected top medical students, the results may not apply to other health professions or medical schools.

## Conclusion

Linking the choice of the class format and learning experiences of preclass online videos sheds light on a step further within a blended framework of precision medical education. Among the three groups (face-to-face, online, or HyFlex), participants who preferred online classes spent the most time online but had lower completion rates for preclass online video learning. Attention should be paid to students who prefer online learning in the design and the practice of HyFlex learning.

Although the three groups had similar short-term learning outcomes, the asynchronous online behavioral characteristics provide potential implications for tailored instructional design in HyFlex learning and strategy development. For example, supplement of interactive elements (discussion, graphs, animations, games, or timelines) in synchronous or asynchronous online learning process might help secure learning engagement among students choosing ‘online only’ class format. To this end, medical institutions may need to invest in faculty training programs and continually adjust to enhance the content of online training and international partnerships [46].

Online learning has allowed medical education to continue and HyFlex design promises learning equity during difficult times. The novelty of our research results can enhance building pro-learning climate and promote learning equity in online medical education. Moving forward, through continuous efforts of improving pedagogical approaches, barriers to online medical education will be minimized to assure quality of medical education.

## Supplementary Material

Supplemental Material

## Data Availability

The datasets used and analyzed during the current study are available from the corresponding author upon reasonable request.

## Declarations

Ethics approval and consent to participate: approval for the collection and publication of student data was obtained from the Institutional Review Board of National Taiwan University Hospital, Taipei, Taiwan. Consent was not obtained from the students because the study was observational, meaning that the students could not be identified from the study data. Therefore, the need for consent was waived by the IRB.
