# Supplementary material for: Linking the choice of the class format and preclass learning experiences sheds light on a step further in blended medical education
Source: Med Educ Online. 2023 Mar 3;28(1):2186207. doi: 10.1080/10872981.2023.2186207 (PMC9987758; doi:10.1080/10872981.2023.2186207)
Supplement: Supplemental Material [file ZMEO_A_2186207_SM3237.docx]

**Supplementary Table S1.** Online Questionnaire Form

**Part I**

**Q1:** Compared with my previous understanding, my knowledge of the core concept (C) after watching the preclass self-learning online video is

C1： □ Totally changed

- Largely changed
- Half and half
- Mostly unchanged
- Totally unchanged

C2： □ Totally changed

- Largely changed
- Half and half
- Mostly unchanged
- Totally unchanged

C3： □ Totally changed

- Largely changed
- Half and half
- Mostly unchanged
- Totally unchanged

C4： □ Totally changed

- Largely changed
- Half and half
- Mostly unchanged
- Totally unchanged

C5： □ Totally changed

- Largely changed
- Half and half
- Mostly unchanged
- Totally unchanged

C6： □ Totally changed

- Largely changed
- Half and half
- Mostly unchanged
- Totally unchanged

**Part II**

**Q2:** Of which learning video(s) I learn most?

□C1, □C2, □C3, □C4, □C5, □C6

**Q3:** I want to learn more about which concept(s)?

□C1, □C2, □C3, □C4, □C5, □C6

**Q4:** I hope the teacher can have more discussions on the topic(s) of

□C1, □C2, □C3, □C4, □C5, □C6

**Q5:** I hope the teacher can delete or decrease discussions on the topic(s) of

□C1, □C2, □C3, □C4, □C5, □C6

**Q6:** If conditions permit, my preferred class format for the upcoming class is

□Face-to-face

□Online

□Both, ‘I want to attend them both’

**Q7:** I feel _____ of the preclass video learning

□Very unsatisfied, □Slight unsatisfied, □Just fine, □Slight satisfied, □Very satisfied

**Part III**

My comment and/or questions___________________________________

**Supplementary Table S2.** Results of self-assessment of preclass learning core concepts and objective assessment of short-term learning outcome.

|  | All | | Face-to face | Online | HyFlex | *P* |
| --- | --- | --- | --- | --- | --- | --- |
| n | 150 | | 71 | 42 | 37 |  |
| Degree of concept agreement (median, IQR)* | | | | | | |
| *C1* | | 4 (3-4) | 4 (3-4) | 4 (3-4) | 4 (3-4) | 0.996 |
| *C2* | | 3 (2-4) | 3 (2-4) | 3 (3-4) | 3 (3-4) | 0.085 |
| *C3* | | 3 (2-4) | 3 (2-4) | 3 (2-4) | 3 (2-4) | 0.214 |
| *C4* | | 3 (2-4) | 3 (2-4) | 3 (2-4) | 3 (2-4) | 0.970 |
| *C5* | | 4 (3-4) | 4 (3-4) | 4 (3-4) | 4 (3-4) | 0.205 |
| *C6* | | 3 (2-4) | 3 (2-4) | 3.5 (2-4) | 3 (2-4) | 0.283 |
| Concept that needs more instruction | | | |  |  |  |
| *C1* | | 22.7 | 17 | 33 | 22 | 0.150 |
| *C2* | | 38.0 | 31 | 45 | 43 | 0.221 |
| *C3* | | 40.0 | 37 | 36 | 51 | 0.271 |
| *C4* | | 59.3 | 55 | 55 | 73 | 0.149 |
| *C5* | | 47.3 | 44 | 52 | 49 | 0.673 |
| *C6* | | 58.7 | 56 | 57 | 65 | 0.683 |
| Concept that do not need further instruction | | | | |  |  |
| *C1* | | 49.3 | 52 | 50 | 43 | 0.674 |
| *C2* | | 13.3 | 17 | 12 | 8 | 0.477 |
| *C3* | | 6.0 | 7 | 10 | 0 | 0.173 |
| *C4* | | 10.7 | 9 | 12 | 14 | 0.622 |
| *C5* | | 10.7 | 13 | 10 | 8 | 0.789 |
| *C6* | | 14.0 | 16 | 19 | 5 | 0.174 |
| Most learnt concept | | |  |  |  |  |
| *C1* | | 36.0 | 30 | 48 | 35 | 0.158 |
| *C2* | | 52.7 | 49 | 57 | 54 | 0.723 |
| *C3* | | 56.0 | 54 | 57 | 60 | 0.849 |
| *C4* | | 58.0 | 56 | 55 | 65 | 0.653 |
| *C5* | | 49.3 | 42 | 55 | 57 | 0.266 |
| *C6* | | 50.7 | 45 | 55 | 57 | 0.434 |
| Objective assessment of performance (mean, SD) | | | | |  |  |
| Case analysis^&^ | | 90.6 (8.5) | 90.3 (11.6) | 90.3 (5.0) | 91.5 (3.4) | 0.609 |
| Assay^$^ | | 2.2 (1.0) | 2.3 (1.0) | 2.1 (1.1) | 2.1 (1.0) | 0.768 |

Data were expressed in percentage unless mentioned otherwise. C, core concept; IQR, interquartile range; SD, standard deviation.

*strongly disagree (1), strongly agree (5); maximum scores of 100(&) and 3($)

**Supplementary Figure S1.** The interrelation of concepts and study context. Survey for information gap after preclass online video learning was back to the teacher and would then guide the teaching in the subsequent synchronous class. Preference of synchronous class format reflects choosing the learning environment which students are comfortable within. Analysis of the link between the choice of the synchronous class format and the previous visualization of preclass videos provides clues of adjusting teaching strategies to facilitate individualized learning process. Noted that the cognitive load had been reduced in designing for the preclass online video learning, as previously described.^28^

**
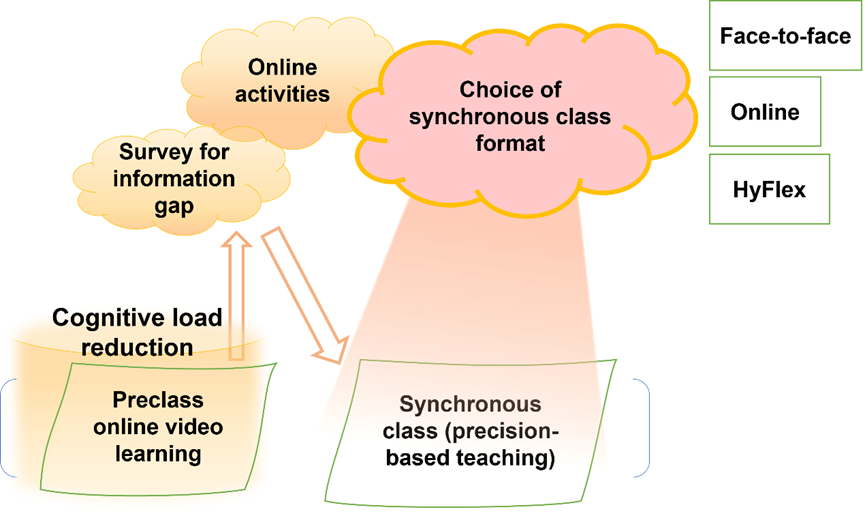
**
